# Comparative *In Vitro* Analysis of Five Starch Sources: Nutrient Release Patterns and Functional Effects in a Simulated Gastrointestinal Environment

**DOI:** 10.3390/foods15081339

**Published:** 2026-04-12

**Authors:** Siyu Yi, Ting Chen, Md. Abul Kalam Azad, Yexing Feng, Pei Wang, Weidong Hu, Qian Zhu, Lei Zhou, Xiangfeng Kong

**Affiliations:** 1 College of Animal Science and Technology, Guangxi University, Nanning 530004, China; 2 Hunan Provincial Key Laboratory of Animal Nutritional Physiology and Metabolic Process, National Engineering Laboratory for Pollution Control and Waste Utilization in Livestock and Poultry Production, Institute of Subtropical Agriculture, Chinese Academy of Sciences, Changsha 410125, China

**Keywords:** biological activities, degradation, free sugars, *in vitro* digestion, metabolites, starch sources

## Abstract

Corn, paddy, wheat, sorghum, and cassava serve as the primary energy sources in both human and animal diets. This study aimed to evaluate their nutrient release patterns in a simulated gastrointestinal environment and to assess the *in vitro* biological activity of the metabolites produced during digestion. The results showed that wheat exhibited the highest dry matter degradation in the stomach–jejunum–ileum digestion stage, while wheat and paddy showed the highest crude protein degradation compared with the other starch sources. In addition, wheat had a higher total free sugar concentration than paddy, sorghum, and cassava. Among the individual free sugars, such as D-sorbitol and D-(+)-trehalose, were found to have the highest concentrations in wheat, whereas cassava had the highest D(−)-fructose concentration. Several differential metabolites, including valeric acid, caproic acid, octanoic acid, and azelaic acid were highly released in paddy, whereas glucaric acid, threonic acid, phenylacetic acid, and shikimic acid were highly released in cassava, and 4-hydroxycinnamic acid was highly released in paddy and sorghum. Four unique metabolites were identified during the digestion process of five starch sources. Particularly, isocitric acid and trans-Cinnamic acid were released only from cassava; caffeic acid was released only from sorghum and corn; and pimelic acid was released only from paddy and wheat. Furthermore, cassava was distinct from the other starch sources, displaying a higher abundance of differential metabolites within the glucagon signaling pathway as mapped in KEGG pathway analysis. In summary, compared with other starch sources, wheat provides more dry matter, protein, and sugars for the body. Cassava is unlikely to offer any advantage in glycemic regulation, while paddy and cassava possess stronger biological activity in terms of differential metabolites.

## 1. Introduction

Corn, paddy, wheat, sorghum, and cassava are common conventional starch sources, mainly providing energy for humans and animals [1,2,3]. Starch is the main component of these crops, typically accounting for approximately 55–85% of their dry weight [2,4,5]. Starch is a crystalline particle composed of two types of glucose polymers, including amylopectin and amylose [6]. Based on digestion speed, starch is classified as rapidly digestible starch (RDS), slowly digestible starch (SDS), and resistant starch [6]. The molecular structure of starch is closely related to its digestion characteristics. For example, the RDS fraction is mainly composed of amylopectin, while amylose is associated primarily with the SDS fraction [4]. Although the research on the digestion characteristics of starch in these starch sources has been extensive, the digestion patterns of the other nutrients present remain an area worth exploring.

Nutrients and metabolites other than starch in cereals and tubers provide various bioactive functions for the body. Previous studies have reported that different starch sources contain distinct bioactive metabolites, such as corn, which is rich in phenols, carotenoids, and anthocyanins; paddy contains phenolic acids, flavonoids, and terpenoids; wheat is generally rich in polyphenols; sorghum contains phenolic acids, anthocyanins, and tannins; and cassava contains hydroxycoumarins, galactosyl diacylglycerides, and β-carotene. These bioactive metabolites exhibit various biological activities, including antioxidant, antibacterial, anti-inflammatory, anti-cancer, and anti-diabetes, etc. [7,8,9,10,11,12]. Collectively, existing research has mainly focused on the metabolites present in the starch sources. However, this approach cannot fully elucidate the actual efficacy of these nutrients and metabolites *in vivo*, due to differences in how different starch sources are digested within the gastrointestinal digestive tract. Moreover, comparative analyses of the nutrient and metabolite profiles among different starch sources are lacking. Therefore, it is urgent to explore the patterns of nutrient release from different starch sources across gastrointestinal segments and thereby clarify their differences.

*In vitro* digestion technology is an advantageous technology to evaluate digestibility, while avoiding the confounding factors associated with *in vivo* nutrient absorption and intestinal motility. At present, most *in vitro* digestion experiments rely on commercial pepsin and pancreatin, which cannot accurately replicate the complex endogenous enzyme profiles of the gastrointestinal tract [4,13,14]. Therefore, to explore the differences in nutrient release patterns, degradation characteristics, and biological activities among various starch sources *in vitro*, we employed a four-stage *in vitro* system using fluid collected from the stomach, jejunum, ileum, and colon of finishing pigs to digest corn, paddy, wheat, sorghum, and cassava. This research provides a novel theoretical basis for advancing the understanding of the biological activity characteristics and gastrointestinal digestion patterns of different starch sources.

## 2. Materials and Methods

### 2.1. Starch Sources and Treatment

Corn was supplied by Haihe Agriculture and Animal Husbandry Technology Co., Ltd., Qingdao, China. Paddy was provided by the Hunan Institute of Agricultural Environment Ecology, Changsha, China. Wheat was obtained from Anhui Fuping Grain Purchase and Sale Co., Ltd., Bozhou, China. Sorghum was sourced from Dongan Xinwufeng Biological Feed Co., Ltd., Yongzhou, China. Cassava was supplied by Tongwei Biotechnology Co., Ltd., Nanning, China. These starch sources were then crushed, passed through a 2 mm sieve, and stored in sealed bags at 4 °C.

### 2.2. Nutritional Composition Analysis

The content of dry matter (DM; method 934.01), organic matter (OM; method 942.05), and crude fat (CF; Method 920.39) was determined in accordance with AOAC methods [15]. The carbon and nitrogen content was analyzed using a carbon and nitrogen element analyzer (Vario MAX cube; Elementar Inc., Hanau, Germany), and crude protein (CP) content was calculated by multiplying the nitrogen content by 6.25. The amylose-to-amylopectin ratio was determined by the amylose/amylopectin assay kit (Megazyme Inc., Bray, Ireland).

The content of neutral detergent fiber (NDF) and acid detergent fiber (ADF) was measured using the methods described by Van Soest et al., [16] with the addition of heat-stable α-amylase (Solarbio Inc., Beijing, China; analytical reagent (AR)) and sodium sulfite (AR) during the NDF analysis. Hemicellulose content was calculated using the following equation:

Hemicellulose (%) = NDF (%) − ADF (%)(1)

Non-fiber carbohydrates (NFC) content was calculated according to Zhang et al. [17] using the following equation:

NFC (%) = 100 − NDF (%) − CP (%) − CF (%)(2)

Calcium and phosphorus content was determined using inductively coupled plasma optical emission spectrometry-5110 (ICP-OES-5110; Agilent Inc., Santa Clara, CA, USA) following high-temperature digestion with nitric acid (AR) and perchloric acid (AR). Gross energy was measured using an isothermal automatic calorimeter (5E-AC8018; Kaide Measurement and Control Instrument Inc., Changsha, China). Hydrolyzed amino acid content was analyzed with an amino acid analyzer (L8900; Hitachi Inc., Tokyo, Japan) after hydrolysis with 6 mol/L hydrochloric acid. All nutritional composition analyses were performed in duplicate.

### 2.3. Collection and Treatment of Digestive Juices

The digestive juices were collected from the stomach, jejunum, ileum, and colon of 16 castrated male Duroc × Landrace × Yorkshire finishing pigs with an average body weight of 123.63 ± 3.12 kg. Pigs were sourced from Hunan New Wellful Co., Ltd., Liuyang, China. All pigs were fasted for 12 h before slaughtering, and the contents of the gastrointestinal tract were collected immediately after slaughter. The contents of the stomach, jejunum, ileum and colon were mixed together, respectively, and then preserved according to the methods previously described by Hamilton et al. [18] and Hu et al. [19]. Briefly, the stomach, jejunum, and ileum contents were immediately centrifuged at 1500× *g* for 10 min at 4 °C, and the supernatants were mixed with 30% glycerol solution at a 1:1 (*v*/*v*) ratio. This glycerol solution contained glycerol (AR) and 0.01 M phosphate-buffered saline (PBS; pH 7.2–7.4; Coolaber Inc., Beijing, China) at a 3:7 (*v*/*v*) ratio. The colonic contents were collected and immediately mixed with 0.01 M PBS solution at a 1:1 (*v*/*v*) ratio, and then filtered through four layers of defatted gauze. The filtered colonic contents were mixed with 30% glycerol solution (*v*/*v* = 3:7 of glycerol (AR) and 0.01 M PBS solution) at a 1:1 (*v*/*v*) ratio. The transportation and handling of gastrointestinal contents were carried out entirely on dry ice. The digestive juices of the stomach, jejunum, ileum, and colon were all stored at −20 °C for 4 h, and then stored at −80 °C. All *in vitro* experiments were completed within 60 days after the collection of digestive juices.

### 2.4. Four-Stage In Vitro Digestion

The procedure and duration of each digestion stage were set according to the methods of Zhu et al. [13] and Du et al. [14] with modifications. For gastric digestion, 1.00 g of each starch source was placed in a 50 mL centrifuge tube with 20 mL of gastric digestive juices (pH adjusted to 2.0 with hydrochloric acid (AR)) and digested in a constant temperature shaker (39 °C and 55 rpm) for 4 h. After gastric digestion, the mixture of undigested residues and gastric juices was centrifuged at 1500× *g* for 10 min at 4 °C. The supernatant was collected and stored at −80 °C for subsequent analysis of free sugars and metabolomics. The remaining undigested residue was then mixed with 20 mL of jejunal digestive juices to continue jejunal digestion in a constant temperature shaker (39 °C and 55 rpm) for 3 h. After this stage, the supernatant was collected after centrifuging at 1500× *g* and 4 °C for 10 min, and the undigested residue was mixed with 20 mL of ileal digestive juices for ileal digestion for 3 h. At the end of ileal digestion, the mixture was centrifuged at 1500× *g* for 10 min at 4 °C to collect the supernatant. The final supernatant was collected, and the undigested residue was filtered into nylon bags, dried at 65 °C for 72 h, and used to calculate DM degradation (DMD), CP degradation (CPD), and carbon degradation (Carbon-D). The remaining undigested residue was used for colonic microbial fermentation *in vitro*. The DMD, CPD, and Carbon-D were calculated using the following equation:

Degradation (%) = (W_0_ − W_t_)/W_0_ × 100. (3)

where W_0_ is the weight of DM, CP, and/or carbon before digestion, and W_t_ is the weight of DM, CP, and/or carbon after digestion. When calculating the DMD of the total intestinal tract, the DM weight of undigested residue was used to determine carbon and CP content after ileal digestion was corrected.

For the colonic fermentation stage, the remaining undigested residue was incubated in 135 mL serum bottles with 48 mL of a buffer solution (containing trace elements and reducing agents [20], pre-charged with CO_2_ at 39 °C for 2 h) and 12 mL of colonic digestive juices. Fermentation proceeded at 39 °C and 55 rpm for 72 h. After colonic digestion, the residue was collected in a nylon bag, dried at 65 °C for 72 h, and analyzed for DMD. All digestion stages were performed in triplicate. A blank control (without a starch source) was included under the same conditions for each experimental run.

### 2.5. Analysis of Free Sugars

The concentrations of free sugars were determined using gas chromatography (7890A; Agilent Inc., Santa Clara, CA, USA) by Biotree Biomedical Technology Co., Ltd., Shanghai, China. Briefly, 1.0 mL of 50% (*v*/*v*) methanol was added to 50 μL of digestive juices or 50 mg of starch sources. The mixture was vortexed for 1 min by a vortex mixer (VXMNAL; Ohaus Inc., New Jersey, NJ, USA), ultrasonicated for 5 min by an ultrasonic cleaner (SB-5200DTD; Xinzhi biotechnology Inc., Ningbo, China), and centrifuged at 12,000× *g* for 5 min to collect supernatant. Subsequently, 0.4 mL of the resulting supernatant was added to a new 1.5 mL Eppendorf (EP) tube and evaporated using an EP concentrator plus (SCIENTZ-10LS; Xinzhi biotechnology Inc., Ningbo, China). The residue was redissolved in 80 μL of trimethoprim solution (20 mg/mL), vortexed for 30 s, and incubated for 60 min at 60 °C. For derivatization, 0.1 mL of N,O-bis(trimethylsilyl) trifluoroacetamide-trimethylchlorosilane (BSTFA-TMCS; 99:1) was added, followed by vortexing for 30 s and incubation at 70 °C for 90 min. Then the sample was centrifuged at 12,000× *g* for 5 min, and approximately 0.1 mL of the supernatant was transferred to a detection vial for analysis. The relative concentration of free sugars was calculated using the following equation:

Relative concentration = Concentration of each treatment group − concentration of blank group.(4)

### 2.6. Metabolomics Analysis

The targeted metabolomics analysis was carried out by Biotree Biomedical Technology Co., Ltd., Shanghai, China. Metabolites were detected by Gas Chromatography-Mass Spectrometry (GC-MS; GC2030-QP2020 NX; Shimadzu Inc., Kyoto, Japan) and Liquid Chromatography-Tandem Mass Spectrometry (LC-MS/MS; 6500+; AB Sciex Inc., Boston, MA, USA) [21,22,23,24,25,26].

For GC-MS analysis, 0.1 mL of digestive juices, 50 µL of 50% (*v*/*v*) sulphuric acid, and 0.2 mL of extracting agent (methyl-tert-butyl ether; AR) were added to a 2 mL EP tube. The mixture was vortexed for 10 s using a vortex mixer (VORTEX-5; Kylin-Bell Lab Instruments Inc., Haimen, China), followed by oscillation for 10 min, sonication for 10 min, and incubation for 10 min in ice water using an ultrasonic cleaner (KS-500DB; Jielimei Ultrasonic Instrument Inc., Kunshan, China). Finally, the sample was centrifuged at 10,000× *g* at 4 °C for 15 min and then placed at −20 °C for 30 min. The supernatant was transferred to a detection vial for GC-MS analysis.

For LC-MS/MS analysis, the sample was first thawed in an ice-water bath and vortexed for 30 s. Then, a 0.1 mL of the sample was mixed with 0.4 mL of methanol (AR) containing an internal standard and vortexed for 30 s. The mixture was sonicated in an ice-water bath for 15 min using an ultrasonic cleaner (KS-500DB; Jielimei Ultrasonic Instrument Inc., Kunshan, China), followed by incubation at −40 °C for 1 h. Afterward, the sample was centrifuged at 12,000× *g* and 4 °C for 15 min to collect supernatant, and 0.2 mL of the supernatant was transferred to a detection vial for LC-MS/MS analysis.

For hierarchical clustering analysis, a Euclidean distance matrix was calculated based on the quantitative values of the top 20 metabolites. These differential metabolites were then clustered using the complete linkage method [27]. The metabolic and regulatory pathways of all differential metabolites were annotated using the Kyoto Encyclopedia of Genes and Genomes (KEGG) database.

### 2.7. Statistical Analysis

The data were analyzed using a linear mixed model in SPSS 26.0 software (SPSS Inc., Chicago, IL, USA). The first analytic model included starch source (*n* = 5) as a fixed effect. When the digestion stage was included, a second model was applied to the relative concentration of free sugars, with starch source (*n* = 5) and the interaction between starch source and digestion stage (*n* = 3) as fixed effects. When a significant interaction was observed, the analysis was repeated for each digestion stage using the first model. Statistically significant differences and trends toward differences were set at the level of *p* < 0.05 and 0.05 ≤ *p* < 0.10, respectively. Principal component analysis (PCA) was performed using a correlation matrix with a 95% confidence interval.

## 3. Results and Discussion

### 3.1. Chemical Compositions of Five Starch Sources

Chemical composition is the main factor affecting the release of nutrients from starch sources in the gastrointestinal tract [28]. For example, amylopectin is primarily associated with the RDS fraction, while amylose is mainly linked to the SDS fraction [4]. In the present study, wheat (0.39) had the highest amylose-to-amylopectin ratio, followed by corn (0.33), paddy (0.32), sorghum (0.30), and cassava (0.24; Table 1). These findings may indicate that the cassava starch contains a higher RDS fraction, while wheat starch has a higher SDS fraction. Compared with SDS, RDS can lead to a rapid increase in blood sugar and insulin levels, which is not conducive to managing obesity, type 2 diabetes, and cardiovascular disease [29]. Thus, we speculated that cassava may be less beneficial for glycemic control compared with the other starch sources.

Fiber content is an important factor limiting dietary digestion, whereas NFC content is positively associated with digestibility [30]. In the present study, paddy contained higher ADF (11.08%) content, while paddy (16.81%) and wheat (15.44%) had higher NDF content. Additionally, cassava (85.21%) and corn (77.13%) had higher NFC content (Table 1). Thus, cassava and corn are likely more digestible, whereas the higher fiber content in paddy and wheat likely inhibits their digestibility.

Protein is a major essential nutrient for the growth, development, and overall health of the body [31]. In the present study, wheat contained the highest CP (16.39%) level and the highest total amino acids (TAA) content (13.22%), followed by sorghum (CP and TAA were 11.91% and 9.49%, respectively; same as below), corn (9.31% and 7.94%), paddy (8.16% and 6.49%), and cassava (2.21% and 1.76%; Table 1 and Table 2). Regarding essential amino acids, wheat had the highest content of isoleucine (0.49%), lysine (0.38%), phenylalanine (0.68%), threonine (0.40%), and valine (0.67%), while sorghum exhibited the highest content of leucine (1.31%; Table 3). Thus, besides supplying energy, wheat plays a vital role in providing higher levels of protein and amino acids, contributing to its nutritional value.

### 3.2. Dry Matter Degradation Characteristics of Five Starch Sources In Vitro

Digestibility is also an important index of dietary nutrition. Previous studies have found that higher fiber content usually reduces the digestibility of starch sources [32,33]. In the present study, wheat exhibited the highest (*p* < 0.05) DMD at the stomach–jejunum–ileum stage, followed by cassava, corn, paddy, and sorghum (Table 3). Notably, although wheat and paddy had higher NDF content, this did not affect their anterior-tract digestibility in the stomach and small intestine. Thus, fiber content may not consistently limit the digestibility of starch sources in the stomach and foregut, and we speculated that such limitation may appear in the fermentation stage of the hindgut.

In the present study, sorghum exhibited a higher (*p* < 0.05) DMD at the colonic stage compared with paddy, wheat, and cassava, while corn had a higher (*p* < 0.05) DMD at the colonic stage compared with paddy and wheat. Furthermore, wheat, corn, and cassava had the highest (*p* < 0.05) total gastrointestinal digestibility, followed by sorghum and paddy (Table 3). The higher content of refractory fiber in paddy may explain its lower degradation in the colon, contributing to its position with the lowest total gastrointestinal digestibility. However, the higher fiber content of wheat did not significantly decrease its digestibility in the whole gastrointestinal tract, possibly due to only a smaller quantity of undigested residues of wheat reaching the colonic fermentation stage. Thus, wheat displayed higher digestibility in both the small intestine and the total gastrointestinal tract, suggesting a more efficient nutrient release pattern.

### 3.3. Crude Protein Degradation Characteristics of Five Starch Sources In Vitro and Their Relationship with Carbon Degradation

**Figure 1:**
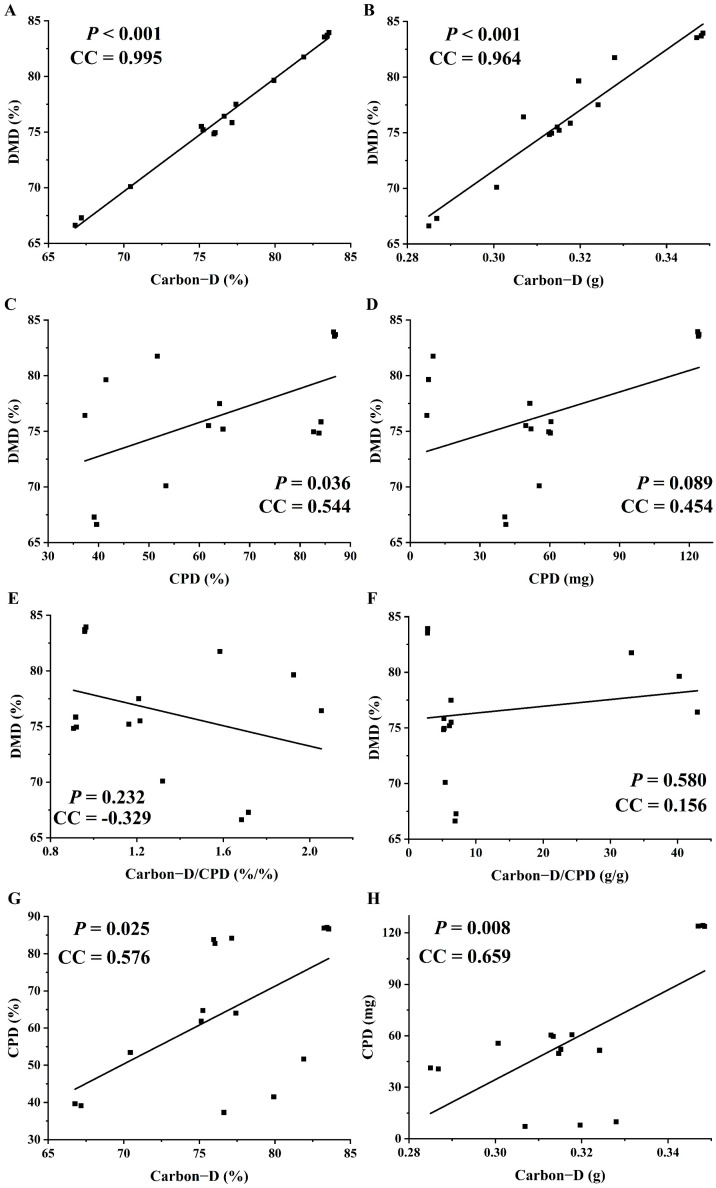
Correlation between degradation characteristics at the stomach–jejunum–ileum digestion stage of five starch sources *in vitro* (*n* = 15). (**A**,**B**) Correlation between DMD and Carbon-D; (**C**,**D**) Correlation between DMD and CPD; (**E**,**F**) Correlation between DMD and Carbon-D/CPD; (**G**,**H**) Correlation between CPD and Carbon-D. DMD, dry matter degradation; CPD, crude protein degradation; Carbon-D, carbon degradation; CC, correlation coefficient.

The protein digestibility is influenced by structural differences during starch degradation [31]. In the present study, paddy and wheat had the highest (*p* < 0.05) CPD at the stomach–jejunum–ileum stage, followed by corn, sorghum, and cassava (Table 3). This order differed from their DMD. In addition, while a positive correlation (*p* < 0.05) existed between CPD and Carbon-D. However, there were no correlations (*p* > 0.10) observed between the Carbon-D/CPD ratio and DMD of different starch sources at the stomach–jejunum–ileum digestion stage (Figure 1). These findings suggested that carbon and nitrogen from the starch sources were not released simultaneously. Compared with the positive correlation (*p* < 0.10) between CPD and DMD, the positive correlation (*p* < 0.001) between Carbon-D and DMD was more relevant (Figure 1). These results indicated that the carbon release in the gastrointestinal tract was accompanied by the degradation of the primary components of the starch sources, whereas protein degradation was more influenced by the structural differences among starch sources. Thus, although some synergy exists between carbon and protein digestion, protein digestibility appears to be more closely related to the intrinsic structure of the protein within the starch source. Moreover, the simultaneous digestibility of carbon and nitrogen was higher in paddy and wheat than in corn, sorghum, and cassava.

### 3.4. Free Sugars Release of Five Starch Sources In Vitro

**Figure 2:**
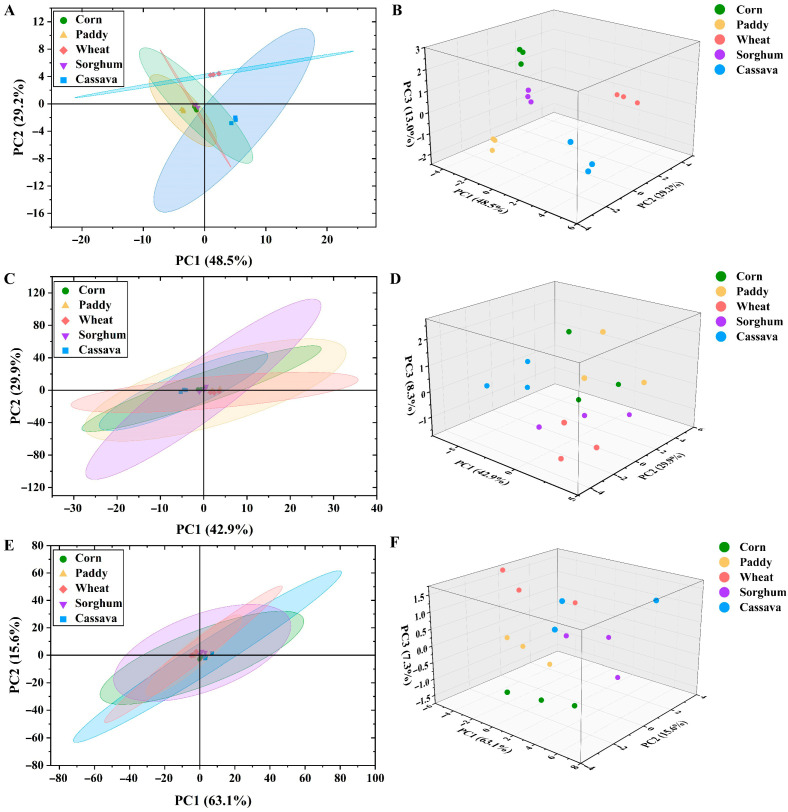
Principal component analysis (PCA) of free sugars of five starch sources *in vitro* digestion (*n* = 3). (**A**,**B**) Stomach stage; (**C**,**D**) Jejunum stage; (**E**,**F**) Ileum stage.

The PCA results showed that the free sugars released from corn and sorghum at the stomach and jejunum (Figure 2A–D) digestion stages were similar. The free sugars released from paddy and wheat at the jejunum stage were similar (Figure 2C,D). Additionally, the free sugars released from five starch sources at the ileum stage were similar (Figure 2E,F). These observations indicate that corn and sorghum have similar patterns of free sugars release in the stomach and small intestine, whereas cassava displays a distinct release pattern compared with the other starch sources.

Starch is mainly digested and absorbed in the small intestine, where it mainly supplies sugars that serve important nutritional functions [34]. In the present study, wheat had the highest (*p* < 0.05) total free sugars concentration in the stomach–jejunum–ileum digestion stage compared with paddy, sorghum, and cassava (Table 4). This finding was consistent with the highest DMD in the small intestine. Furthermore, the concentration of total free sugars was highest (*p* < 0.05) at the jejunum digestion stage, followed by the ileum and stomach (Table 4). This finding indicates that the primary degradation of starch occurred in the jejunum, and the least degradation occurred in the stomach.

Dietary polysaccharides cannot be easily digested in the stomach due to the lack of specific digestive enzymes [35]. In the present study, cassava had the highest concentration of total free sugars (1.08 mg/g), followed by wheat (1.04 mg/g), corn and sorghum (0.87 mg/g), and paddy (0.64 mg/g; Table S1). As for the digestive juices, cassava also had the highest concentration of total free sugars (*p* < 0.05), followed by wheat, corn, sorghum, and paddy (Table S2). These findings were consistent with the results for total free sugars of these starch sources. Thus, we speculated that most of the free sugars released by starch sources in the stomach stage may originate from the free sugars carried by themselves.

Numerous free sugars released by starch sources usually have some unique effects. For example, galactose, mannose, sorbitol, and trehalose play significant roles in regulating glycemia and immunity, inhibiting tumors and inflammatory diseases, etc. [36,37,38,39,40,41]. However, excessive fructose intake has been reported to be associated with an increased risk of chronic diseases such as obesity, nonalcoholic fatty liver disease, and cardiovascular disease [42]. In the present study, wheat had the highest (*p* < 0.05) concentrations of D-sorbitol and D-(+)-trehalose; cassava had the highest (*p* < 0.05) concentrations of D(−)-fructose; and wheat and cassava had the highest (*p* < 0.05) concentrations of D-galactose and D-(+)-mannose in the stomach–jejunum–ileum digestion stage (Table 4). Therefore, wheat may exhibit more bioactive functions for glycemia regulation compared to other starch sources, and the higher fructose released by cassava may make it unsuitable for excessive intake.

### 3.5. Differences in Metabolites and Their KEGG Pathways and Functions of Five Starch Sources In Vitro

**Figure 3:**
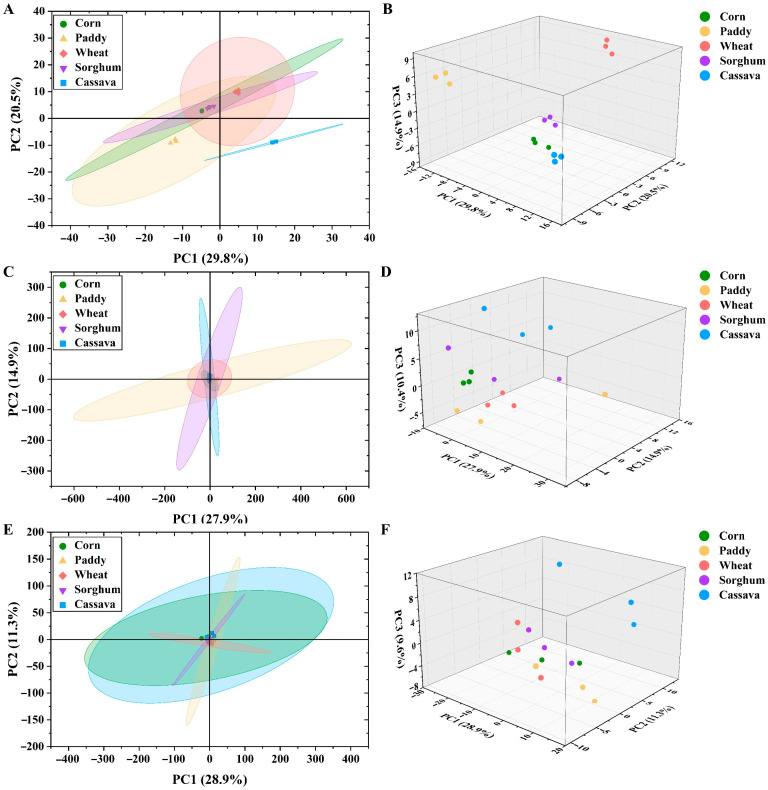
Principal component analysis (PCA) of metabolites of five starch sources *in vitro* digestion (*n* = 3). (**A**,**B**) Stomach stage; (**C**,**D**) Jejunum stage; (**E**,**F**) Ileum stage.

**Figure 4:**
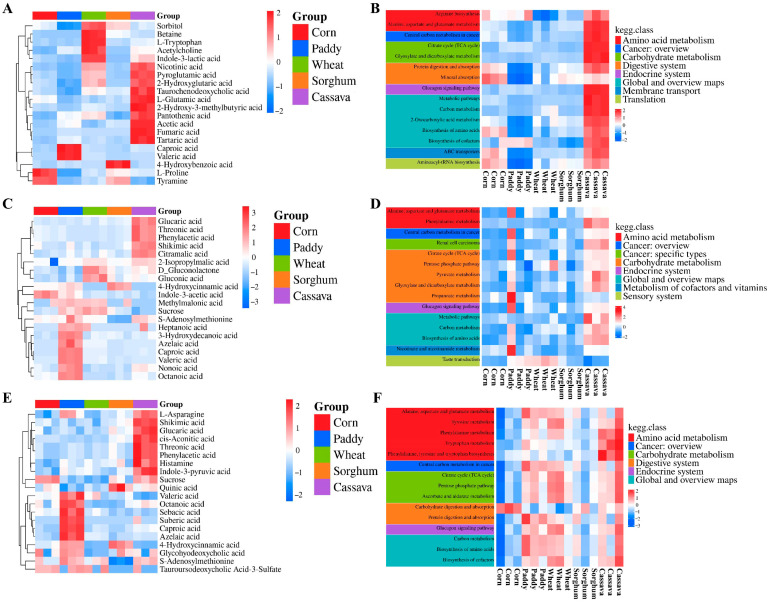
Heatmap of hierarchical clustering analysis (the top 20) and Kyoto Encyclopedia of Genes and Genomes (KEGG; the top 15) heatmap of differential metabolites of five starch sources *in vitro* digestion (*n* = 3). (**A**,**B**) Stomach stage; (**C**,**D**) Jejunum stage; (**E**,**F**) Ileum stage.

Similar to the free sugars results, the metabolomics PCA results revealed that corn and sorghum had a similar pattern at the stomach stage. Cassava displayed a different metabolite composition at the stomach, jejunum, and ileum stages compared with the other starch sources (Figure 3A,D,F). The difference between cassava and the other starch sources was also further reflected in the KEGG pathway analysis of differential metabolites. Notably, cassava was highly enriched in several pathways at the stomach, jejunum, and ileum digestion stages, which mainly included alanine/aspartate/glutamate metabolism, central carbon metabolism in cancer, glucagon signaling pathways, etc. Paddy and wheat were highly enriched in the taste transduction pathway at the jejunum stage. Moreover, at the ileum stage, paddy, wheat, and cassava were highly enriched in the central carbon metabolism in cancer, glucagon signaling pathways, and others. (Figure 4B,D,F). Taken together, these results suggest that the overall metabolic function of cassava may be higher than that of corn and sorghum. Furthermore, we speculated that corn and sorghum may have a higher potential to regulate glycemia than cassava.

In the present study, paddy released higher (*p* < 0.05) concentrations of valeric acid and caproic acid at the stomach, jejunum, and ileum digestion stages (Figure 4A,C,E). At the jejunum and ileum stages, paddy also released higher (*p* < 0.05) concentrations of octanoic acid and azelaic acid. Cassava released higher (*p* < 0.05) concentrations of glucaric acid, threonic acid, phenylacetic acid, and shikimic acid at the jejunum and ileum stages. Paddy and sorghum released higher (*p* < 0.05) 4-hydroxycinnamic acid concentration, at the jejunum and ileum stages (Figure 4C,E). These metabolites are associated with potential bioactive properties. For example, valeric acid, octanoic acid, azelaic acid, glucaric acid, shikimic acid, and phenylacetic acid have been associated with neuroprotection, anti-cancer, anti-diabetes, anti-hypertension, and anti-inflammatory effects [43,44,45,46,47,48,49,50,51,52]. Caproic acid aids in regulating the inflammatory response [53], and 4-hydroxycinnamic acid has a higher antioxidant potential [54]. Therefore, paddy and cassava may have higher biological activity and significant potential in combating several diseases due to their bioactive components compared with other starch sources.

**Figure 5:**
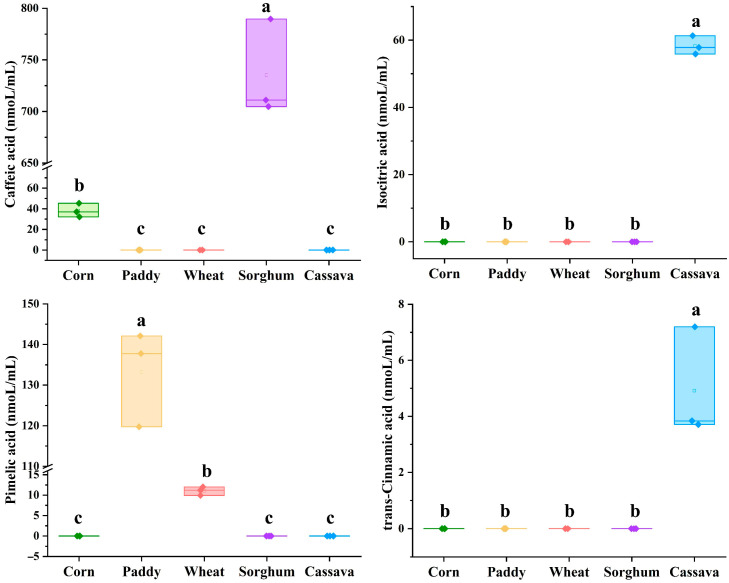
The concentration of specific metabolites (only found in two or less starch sources) of five starch sources at the stomach–jejunum–ileum digestion stage *in vitro*. Different letters indicate significant differences (*p* < 0.05) between different groups (*n* = 3).

Four unique metabolites were released from five starch sources. These metabolites included isocitric acid and trans-Cinnamic acid from cassava, while caffeic acid was from sorghum and corn. Additionally, the concentration of caffeic acid was higher (*p* < 0.05) in sorghum than in corn. Pimelic acid was only released from paddy and wheat, while the concentration of pimelic acid was higher (*p* < 0.05) in paddy than in wheat (Figure 5). Isocitric acid is a key intermediate metabolite of the citrate cycle in organisms and plays a key role in energy production and metabolic regulation. The higher isocitrate concentration released from cassava was consistent with its higher enrichment in the citrate cycle pathway observed for that starch source. Trans-Cinnamic acid and pimelic acid have strong antifungal activity, as well as antibacterial and antifatigue activities, respectively [48,55,56]. Caffeic acid is associated with potential anti-inflammatory, antiviral, anti-cancer, anti-diabetes, antibacterial, neuroprotective, and hepatoprotective effects [57,58,59]. Therefore, the higher trans-Cinnamic acid content in cassava, pimelic acid in paddy, and caffeic acid in sorghum may contribute to their enhanced effectiveness against harmful microbial invasion. Moreover, the biological significance of these unique metabolites during human digestion and metabolism warrants further investigation.

## 4. Conclusions

Wheat exhibited a superior nutrient release pattern, characterized by higher CP and amino acid content, enhanced DMD and CPD, as well as better simultaneous digestibility of carbon and nitrogen. Wheat also contained higher concentrations of D-sorbitol and D-(+)-trehalose, indicating potential biologically effectiveness in regulating glycemia. However, cassava intake was presumed not to be conducive to glycemia regulation. Several distinct differential metabolites were identified in different starch sources, such as valeric acid and pimelic acid in paddy, glucaric acid and trans-Cinnamic acid in cassava, and 4-hydroxycinnamic acid and caffeic acid in sorghum. Future research should focus on exploring the practical application of these bioactive components derived from different starch sources.

## Figures and Tables

**Table 1:** Nutritional composition of five starch sources (%, as dry matter basis).

Item	Corn	Paddy	Wheat	Sorghum	Cassava
OM	98.85	95.60	98.05	98.59	97.73
Amylose-to-amylopectin ratio	0.33	0.32	0.39	0.30	0.24
Carbon	48.51	46.66	47.90	48.93	46.24
CP	9.31	8.16	16.39	11.91	2.21
CF	3.56	1.84	1.35	3.74	0.80
NDF	10.00	16.81	15.44	12.04	11.78
ADF	0.49	11.08	2.67	1.84	2.79
Hemicellulose	9.52	5.73	12.78	10.20	8.10
NFC	77.13	73.19	66.82	72.31	85.21
Ca	0.10	0.54	0.34	0.18	1.11
P	2.80	2.81	3.85	2.86	0.73
GE	19.48	18.94	19.48	19.83	18.09

OM, organic matter; CP, crude protein; CF, crude fat; NDF, neutral detergent fiber; ADF, acid detergent fiber; NFC, non-fiber carbohydrates; Ca, calcium, mg/g of DM; P, phosphorus, mg/g of DM; GE, gross energy, MJ/Kg of DM.

**Table 2:** Hydrolyzed amino acid composition of five starch sources (%, as dry matter basis).

Item	Corn	Paddy	Wheat	Sorghum	Cassava
Essential amino acids					
Histidine	0.25	0.18	0.34	0.23	0.04
Isoleucine	0.28	0.28	0.49	0.40	0.08
Leucine	1.01	0.57	0.95	1.31	0.23
Lysine	0.26	0.27	0.38	0.23	0.04
Phenylalanine	0.42	0.37	0.68	0.54	0.10
Threonine	0.31	0.27	0.40	0.33	0.06
Total	2.55	1.95	3.25	3.04	0.56
Nonessential amino acids					
Alanine	0.63	0.43	0.56	0.93	0.17
Arginine	0.40	0.52	0.67	0.38	0.08
Aspartic acid	0.57	0.65	0.70	0.68	0.12
Glutamate	1.59	1.25	4.28	2.05	0.37
Glycine	0.34	0.34	0.61	0.34	0.06
Proline	0.79	0.37	1.53	0.83	0.16
Serine	0.39	0.33	0.60	0.43	0.08
Tyrosine	0.27	0.21	0.35	0.30	0.07
Valine	0.40	0.44	0.67	0.51	0.10
Total	5.39	4.54	9.97	6.45	1.23
Total amino acids	7.94	6.49	13.22	9.49	1.76

**Table 3:** Degradation characteristics of five starch sources *in vitro* (%, as dry matter basis).

Item	Corn	Paddy	Wheat	Sorghum	Cassava	SEM	*p*-Values
Stomach–jejunum–ileum stage							
DMD	76.07 ^c^	75.21 ^c^	83.73 ^a^	68.00 ^d^	79.27 ^b^	1.426	<0.001
Carbon-D	75.93 ^c^	76.38 ^c^	83.43 ^a^	68.13 ^d^	79.49 ^b^	1.393	<0.001
CPD	63.55 ^b^	83.54 ^a^	86.85 ^a^	44.06 ^c^	43.49 ^c^	5.076	<0.001
Carbon-D/CPD	1.20 ^c^	0.91 ^d^	0.96 ^cd^	1.57 ^b^	1.86 ^a^	0.102	<0.001
Colonic stage							
DMD	12.99 ^ab^	0.92 ^d^	5.60 ^c^	14.21 ^a^	9.36 ^bc^	1.390	<0.001
Total gastrointestinal tract							
DMD	89.06 ^a^	76.13 ^c^	89.33 ^a^	82.21 ^b^	88.63 ^a^	1.426	<0.001

DMD, dry matter degradation; CPD, crude protein degradation; Carbon-D, carbon degradation. Different lowercase superscript letters within the same row indicate significant differences (*p* < 0.05) between different groups (*n* = 3).

**Table 4:** The relative concentrations of free sugars at the stomach–jejunum–ileum digestion stage of five starch sources *in vitro* (μg/mL).

Item	Starch Source					Digestion Stage			SEM	*p*-Values		
	Corn	Paddy	Wheat	Sorghum	Cassava	Stomach	Jejunum	Ileum		Source	Stage	Source × Stage
L-Arabinose	1.10 ^a^	0.69 ^c^	1.04 ^a^	0.93 ^ab^	0.73 ^bc^	1.12 ^B^	1.37 ^A^	0.19 ^C^	0.100	0.003	<0.001	<0.001
D(−)-Fructose	20.98 ^bc^	9.02 ^d^	25.83 ^b^	15.38 ^cd^	115.96 ^a^	97.95 ^A^	13.00 ^B^	1.35 ^C^	11.446	<0.001	<0.001	<0.001
L-(−)-Fucose	−1.06	−0.98	−0.93	−0.52	−0.87	0.14 ^A^	0.09 ^A^	−2.85 ^B^	0.225	0.220	<0.001	0.195
D-Galactose	26.60 ^c^	23.95 ^d^	30.90 ^a^	27.83 ^b^	30.36 ^a^	14.64 ^C^	35.39 ^A^	33.74 ^B^	1.594	<0.001	<0.001	<0.001
D-Galaturonic acid	0.85 ^a^	0.75 ^b^	0.62 ^c^	0.74 ^b^	0.74 ^b^	0.45 ^B^	1.43 ^A^	0.34 ^C^	0.078	0.001	<0.001	<0.001
D-Glucose	4522.54 ^a^	4452.54 ^ab^	4576.32 ^a^	4266.67 ^bc^	4187.45 ^c^	271.01 ^C^	8947.06 ^A^	3985.24 ^B^	547.926	0.002	<0.001	<0.001
D-Glucose-6-phosphate	52.90	58.60	56.32	56.94	55.79	0.17 ^C^	86.79 ^A^	81.37 ^B^	6.100	0.204	<0.001	<0.001
D-Glucuronic acid	0.17 ^b^	0.06 ^d^	0.11 ^c^	0.12 ^c^	0.30 ^a^	0.27 ^A^	0.15 ^B^	0.05 ^C^	0.025	<0.001	<0.001	<0.001
Inositol	4.08 ^bc^	2.56 ^c^	4.17 ^bc^	5.73 ^b^	7.79 ^a^	22.53 ^A^	1.76 ^B^	−1.27 ^C^	2.489	<0.001	<0.001	<0.001
Lactose	1.53 ^bc^	1.78 ^a^	1.77 ^a^	1.56 ^b^	1.35 ^c^	0.05 ^C^	2.92 ^A^	1.84 ^B^	0.194	<0.001	<0.001	<0.001
Maltose	2.53 ^c^	2.12 ^c^	31.79 ^a^	2.76 ^c^	6.22 ^b^	22.18 ^A^	4.12 ^B^	0.95 ^C^	3.258	<0.001	<0.001	<0.001
D-(+)-Mannose	26.30 ^b^	23.43 ^c^	30.38 ^a^	27.00 ^b^	31.45 ^a^	13.39 ^C^	32.49 ^B^	37.26 ^A^	1.755	<0.001	<0.001	<0.001
Raffinose	3.19 ^a^	1.97 ^bc^	1.51 ^c^	3.53 ^a^	2.68 ^ab^	6.53 ^A^	1.13 ^B^	0.06 ^C^	0.476	0.001	<0.001	0.001
L-Rhamnose	0.63	0.08	−0.41	1.10	−0.12	0.37	1.09	−0.70	0.312	0.572	0.081	0.732
D-Sorbitol	18.69 ^c^	4.12 ^e^	43.36 ^a^	27.60 ^b^	15.08 ^d^	60.72 ^A^	4.25 ^B^	0.35 ^C^	5.306	<0.001	<0.001	<0.001
Sucrose	10.68 ^a^	6.34 ^d^	7.76 ^c^	9.22 ^b^	10.62 ^ab^	26.75 ^A^	0.26 ^B^	−0.24 ^B^	1.957	<0.001	<0.001	<0.001
D-(+)-Trehalose	1.29 ^c^	1.60 ^c^	12.55 ^a^	1.28 ^c^	2.50 ^b^	8.50 ^A^	2.40 ^B^	0.62 ^C^	1.222	<0.001	<0.001	<0.001
Trehalose-6-phosphate	14.57 ^b^	28.33 ^a^	29.19 ^a^	11.96 ^bc^	9.24 ^c^	0.09 ^C^	49.11 ^A^	6.76 ^B^	4.102	<0.001	<0.001	<0.001
Xylose	1.13	1.15	1.16	1.24	1.16	0.19 ^C^	2.06 ^A^	1.26 ^B^	0.122	0.900	<0.001	0.035
Total free sugars	4708.67 ^ab^	4618.09 ^bc^	4853.43 ^a^	4461.05 ^c^	4492.48 ^c^	547.05 ^C^	9189.87 ^A^	4146.31 ^B^	547.626	0.003	<0.001	<0.001

Different lowercase superscript letters within the same row indicate significant differences (*p* < 0.05) between different groups in the treatment of starch sources; different uppercase superscript letters indicate significant differences (*p* < 0.05) between different groups in the treatment of digestion stage (*n* = 3).

## Data Availability

The original contributions presented in this study are included in the article/Supplementary Material. Further inquiries can be directed to the corresponding authors.

## Supplementary Materials

The following supporting information can be downloaded at: https://www.mdpi.com/article/10.3390/foods15081339/s1. Table S1: The composition of free sugars in five starch sources (μg/g, as DM basis). Table S2: The relative concentrations of free sugars in five starch sources at the stomach digestion stage *in vitro* (μg/mL). Table S3: The relative concentrations of free sugars in five starch sources at the jejunum digestion stage *in vitro* (μg/mL). Table S4: The relative concentrations of free sugars in five starch sources at the ileum digestion stage of five starch sources *in vitro* (μg/mL).
