# Supplementary material for: Comparative In Vitro Analysis of Five Starch Sources: Nutrient Release Patterns and Functional Effects in a Simulated Gastrointestinal Environment
Source: Foods. 2026 Apr 12;15(8):1339. doi: 10.3390/foods15081339 (PMC13114331; doi:10.3390/foods15081339)
Supplement: Supplementary file 1 [file foods-15-01339-s001.zip › foods-4223795-supplementary.pdf]

Table S1. The composition of free sugars in five starch sources (μg/g, as DM basis).

Item	Corn	Paddy	Wheat	Sorghum	Cassava
L-Arabinose	32.71	3.70	24.23	9.70	36.72
D(-)-Fructose	149.01	86.68	114.38	94.48	90.19
L-(-)-Fucose	0.83	3.83	0.93	0.73	8.07
D-Galactose	70.62	65.45	98.04	92.44	56.34
D-Galaturonic acid	2.06	0.98	1.01	1.35	0.56
D-Glucose	72.40	66.40	65.73	73.96	41.45
D-Glucose-6-phosphate	1.36	0.83	10.93	1.24	68.06
D-Glucuronic acid	15.92	0.42	2.78	7.51	21.16
Inositol	48.84	32.88	58.36	53.82	72.94
Lactose	18.49	1.55	14.69	4.61	69.65
Maltose	72.29	45.14	263.60	103.09	201.52
D-(+)-Mannose	73.85	68.43	85.53	96.64	47.45
Raffinose	114.18	88.07	56.36	102.19	87.23
L-Rhamnose	7.47	7.57	9.36	8.75	10.65
D-Sorbitol	95.83	60.36	120.45	125.71	108.43
Sucrose	37.06	36.91	29.38	23.98	39.15
D-(+)-Trehalose	47.74	69.82	72.85	50.60	84.78
Trehalose-6-phosphate	3.45	1.54	3.03	15.53	19.60
Xylose	3.38	1.38	9.73	2.49	18.55
Total free sugars	867.49	641.92	1041.39	868.80	1082.51

Table S2. The relative concentrations of free sugars in five starch sources at the stomach digestion stage *in vitro* (μg/mL).

**Item**	**Corn**	**Paddy**	**Wheat**	**Sorghum**	**Cassava**	**SEM**	***P*-value**
L-Arabinose	1.49^{b}	0.34^{d}	1.66^{a}	1.03^{c}	1.08^{c}	0.124	< 0.001
D(-)-Fructose	51.67^{bc}	21.61^{d}	68.78^{b}	37.35^{cd}	310.37^{a}	28.834	< 0.001
D-Galactose	9.40^{d}	3.23^{e}	25.58^{a}	13.84^{c}	21.15^{b}	2.177	< 0.001
D-Galaturonic acid	0.57^{b}	0.25^{e}	0.35^{d}	0.42^{c}	0.66^{a}	0.040	< 0.001
D-Glucose	177.69^{b}	78.40^{c}	148.44^{b}	140.86^{b}	809.66^{a}	72.855	< 0.001
D-Glucose-6-phosphate	-0.03^{c}	-0.04^{c}	0.16^{b}	-0.01^{c}	0.76^{a}	0.082	< 0.001
D-Glucuronic acid	0.32^{b}	0.01^{d}	0.15^{c}	0.17^{c}	0.70^{a}	0.063	< 0.001
Inositol	11.67^{bc}	7.20^{c}	12.74^{b}	15.31^{b}	66.74^{a}	5.854	< 0.001
Lactose	0.03^{bc}	-0.02^{d}	0.15^{a}	-0.01^{cd}	0.07^{b}	0.017	< 0.001
Maltose	2.96^{c}	0.30^{c}	88.56^{a}	3.65^{c}	15.46^{b}	9.007	< 0.001
D-(+)-Mannose	7.31^{bc}	2.61^{c}	26.01^{a}	10.24^{b}	20.78^{a}	2.420	< 0.001
Raffinose	8.23^{a}	4.77^{b}	3.91^{b}	9.10^{a}	6.66^{ab}	0.640	0.015
D-Sorbitol	52.27^{c}	10.27^{e}	122.87^{a}	76.45^{b}	41.73^{d}	10.106	< 0.001
Sucrose	32.90^{a}	19.88^{c}	24.16^{b}	25.55^{b}	31.25^{a}	1.338	< 0.001
D-(+)-Trehalose	1.06^{c}	0.94^{c}	33.62^{a}	1.30^{c}	5.58^{b}	3.402	< 0.001
Trehalose-6-phosphate	0.02^{b}	0.06^{b}	0.11^{ab}	0.08^{b}	0.19^{a}	0.019	0.043
Xylose	0.10^{c}	0.05^{d}	0.24^{b}	0.10^{c}	0.47^{a}	0.042	< 0.001
Total free sugars	357.89^{c}	150.25^{d}	558.07^{b}	335.95^{c}	1333.08^{a}	111.094	< 0.001

Different letters indicate significant differences ($P < 0.05$) between different groups ($n = 3$).

Table S3. The relative concentrations of free sugars in five starch sources at the jejunum digestion stage *in vitro* (μg/mL).

Item	Corn	Paddy	Wheat	Sorghum	Cassava	SEM	*P*-value
L-Arabinose	1.74^{a}	1.63^{a}	1.43^{a}	1.55^{a}	0.53^{b}	0.140	0.015
D(-)-Fructose	11.17^{b}	5.55^{d}	8.69^{c}	8.55^{c}	31.04^{a}	2.466	< 0.001
D-Galactose	35.68^{a}	36.16^{a}	35.18^{ab}	35.76^{a}	34.18^{b}	0.232	0.032
D-Galaturonic acid	1.59^{a}	1.68^{a}	1.24^{b}	1.41^{ab}	1.23^{b}	0.061	0.021
D-Glucose	8789.28^{b}	9900.62^{a}	10318.11^{a}	8578.73^{b}	7148.58^{c}	304.08	< 0.001
D-Glucose-6-phosphate	72.70^{c}	101.38^{a}	95.76^{ab}	85.77^{bc}	78.35^{c}	3.369	0.009
D-Glucuronic acid	0.15	0.15	0.15	0.15	0.15	0.003	0.979
Inositol	2.28	2.30	1.31	2.43	0.48	0.321	0.239
Lactose	2.52^{b}	3.80^{a}	3.70^{a}	2.83^{b}	1.73^{c}	0.218	< 0.001
Maltose	3.35^{b}	5.49^{a}	6.33^{a}	3.67^{b}	1.75^{c}	0.453	< 0.001
D-(+)-Mannose	33.26^{ab}	31.55^{bc}	29.79^{c}	33.53^{a}	34.31^{a}	0.483	0.001
Raffinose	1.31^{a}	1.17^{a}	0.67^{b}	1.30^{a}	1.21^{a}	0.076	0.013
D-Sorbitol	3.56^{b}	2.47^{b}	6.57^{a}	5.82^{a}	2.80^{b}	0.475	< 0.001
Sucrose	0.64	0.35	0.13	-0.12	0.31	0.110	0.280
D-(+)-Trehalose	2.06^{b}	3.39^{a}	3.59^{a}	1.88^{b}	1.09^{c}	0.259	< 0.001
Trehalose-6-phosphate	34.26^{b}	81.20^{a}	83.73^{a}	29.25^{b}	17.11^{c}	7.551	< 0.001
Xylose	2.00	2.27	2.15	2.23	1.64	0.112	0.427
Total free sugars	9000.57^{b}	10182.01^{a}	10598.05^{a}	8797.74^{b}	7355.97^{c}	311.85	< 0.001

Different letters indicate significant differences ($P < 0.05$) between different groups ($n = 3$).

Table S4. The relative concentrations of free sugars in five starch sources at the ileum digestion stage of five starch sources *in vitro* (μg/mL).

Item	Corn	Paddy	Wheat	Sorghum	Cassava	SEM	*P*-value
L-Arabinose	0.06^{b}	0.09^{b}	0.04^{b}	0.20^{b}	0.58^{a}	0.068	0.026
D(-)-Fructose	0.10^{b}	-0.09^{b}	0.00^{b}	0.24^{b}	6.48^{a}	0.690	< 0.001
D-Galactose	34.71^{ab}	32.46^{c}	31.94^{c}	33.88^{b}	35.74^{a}	0.401	< 0.001
D-Galaturonic acid	0.39	0.32	0.26	0.40	0.34	0.018	0.082
D-Glucose	4600.64^{a}	3378.61^{c}	3262.41^{c}	4080.43^{b}	4604.12^{a}	161.297	< 0.001
D-Glucose-6-phosphate	86.04^{a}	74.45^{b}	73.03^{b}	85.06^{a}	88.27^{a}	1.757	< 0.001
D-Glucuronic acid	0.05^{b}	0.03^{c}	0.03^{c}	0.05^{b}	0.06^{a}	0.003	0.002
Inositol	-1.71	-1.83	-1.54	-0.56	-0.72	0.214	0.180
Lactose	2.05^{ab}	1.55^{c}	1.46^{c}	1.87^{b}	2.25^{a}	0.085	< 0.001
Maltose	1.26^{a}	0.57^{c}	0.50^{c}	0.95^{b}	1.45^{a}	0.103	< 0.001
D-(+)-Mannose	38.33^{a}	36.14^{c}	35.33^{c}	37.22^{b}	39.28^{a}	0.399	< 0.001
Raffinose	0.03^{ab}	-0.03^{b}	-0.06^{b}	0.20^{a}	0.19^{a}	0.038	0.048
D-Sorbitol	0.23^{b}	-0.38^{c}	0.64^{a}	0.53^{a}	0.72^{a}	0.113	< 0.001
Sucrose	-1.51	-1.21	-1.01	2.24	0.29	0.520	0.104
D-(+)-Trehalose	0.74^{ab}	0.47^{c}	0.46^{c}	0.64^{b}	0.81^{a}	0.040	< 0.001
Trehalose-6-phosphate	9.41^{a}	3.72^{c}	3.72^{c}	6.54^{b}	10.42^{a}	0.794	< 0.001
Xylose	1.28^{ab}	1.15^{bc}	1.09^{c}	1.40^{a}	1.37^{a}	0.039	0.015
Total free sugars	4767.55^{a}	3522.02^{c}	3404.15^{c}	4249.46^{b}	4788.39^{a}	165.242	< 0.001

Different letters indicate significant differences ($P < 0.05$) between different groups ($n = 3$).
